# Churning-Motion-Assisted Bubble Removal: A Low-Cost Approach for Enhancing PDMS Mixture Quality

**DOI:** 10.1021/acsomega.4c05290

**Published:** 2024-08-02

**Authors:** Anoop Kanjirakat, Naresh Kumar Mani, Dolfred Vijay Fernandes

**Affiliations:** † Department of Aeronautical & Automobile Engineering, Manipal Institute of Technology, Manipal Academy of Higher Education, Manipal, 576104, India; ‡ Microfluidics, Sensors and Diagnostics (μSenD) Laboratory, Centre for Microfluidics, Biomarkers, Photoceutics and Sensors (μBioPS), Department of Biotechnology, Manipal Institute of Technology, Manipal Academy of Higher Education, Manipal 576104, Karnataka, India; § Department of Mechanical & Industrial Engineering, Manipal Institute of Technology, Manipal Academy of Higher Education, Manipal, 576104, India

## Abstract

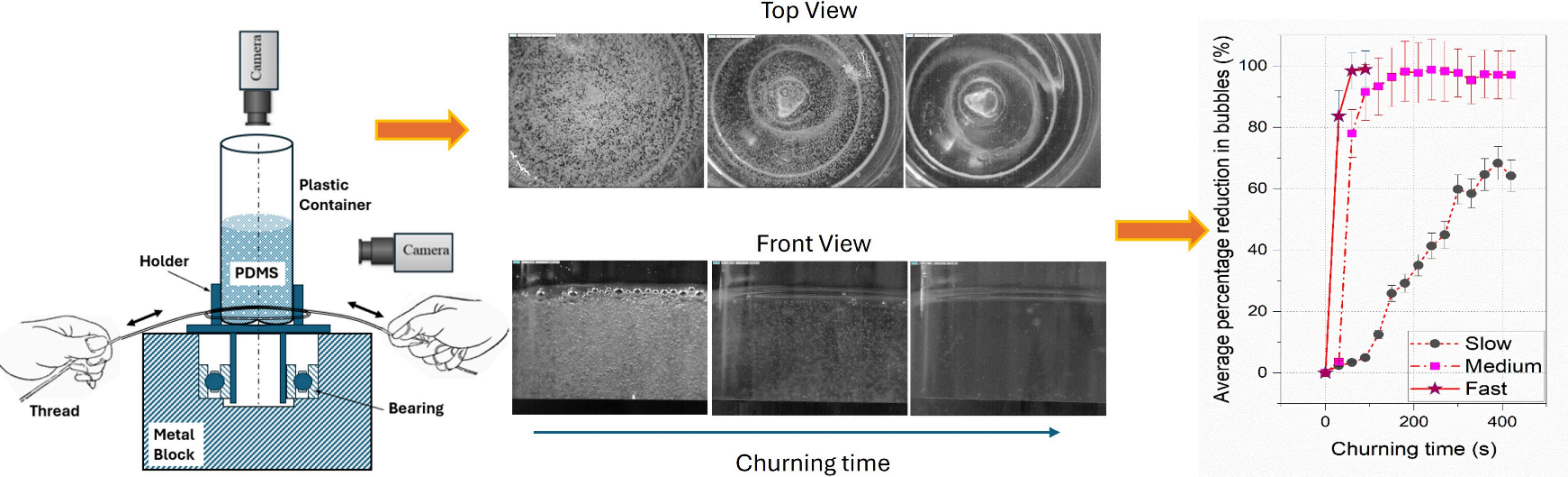

Bubble formation during mixing of the base elastomer and the curing agent for polydimethylsiloxane (PDMS) preparation presents a significant challenge, traditionally addressed through vacuum degassing or centrifugation. This study introduces a novel alternative for bubble removal in PDMS mixtures: a churning motion inspired by industrial dairy separation processes. A low-cost, manually operated, do-it-yourself (DIY) churning device has been developed for this purpose. We investigate the effectiveness of churning in eliminating bubbles across three different churning speeds and two PDMS mixtures with differing viscosities. The efficacy of this method is quantitatively assessed through the analysis of images captured during the churning process. The results demonstrate that bubble removal is notably more efficient in the PDMS mixture with a higher viscosity due to enhanced bubble coalescence. Among the tested speeds, fast churning emerges as the most effective, achieving bubble removal in less than 100 s—significantly outperforming the traditional vacuum degassing method, which requires over 3000 s. These findings highlight churning motion as a rapid, efficient, and cost-effective alternative for bubble removal in PDMS processing, promising significant advancements in material preparation techniques.

## 1 Introduction

Polydimethylsiloxane (PDMS) based microchannels are integral components in the field of microfluidics due to their outstanding properties, which include optical transparency, biocompatibility, elasticity, flexibility, and low surface adsorption.^1^ The soft lithography technique allows for the rapid and cost-effective fabrication of PDMS microchannels with intricate geometries, facilitating the integration of multiple functionalities within a single device.^2^

Typical preparation of PDMS involves thoroughly mixing the base polymer and curing agent in the correct proportion to ensure homogeneity. PDMS elastomer base is made of polydimethylsiloxane chains synthesized from dimethyldichlorosilane or tetramethyl-disiloxane hydrolysis.^3^ The elastomer nature of PDMS is due to these chains providing flexibility, stretchability, and resilience. The curing agent, also known as a cross-linker, contains multifunctional siloxane with either vinyl or hydrosilane functionalities.^4^ When mixed with the elastomer base, the curing agent helps with cross-linking, creating a three-dimensional network of polymer chains within the PDMS matrix.^5^ This cross-linking process is essential for turning the liquid elastomer mix into a solid, flexible material, and it is commonly initiated by heat or a catalyst. The ratio of elastomer base to curing agent decides the mechanical properties and curing time of the PDMS. Higher curing agent concentrations make the material stiffer and require a shorter curing time.^6^ The choice of curing conditions, such as temperature and duration, can also affect the final properties of the PDMS.

One unavoidable complexity in the mixing process is the formation of bubbles in the PDMS mixture. The trapped bubbles can have detrimental effects on the material properties and functionality of the final PDMS sample. The foremost concern is where optical clarity is paramount, such as in microfluidic devices, where bubbles can significantly hinder performance. This is due to the scattering of light caused by bubbles, resulting in decreased optical resolution and clarity.^4^ The scattering effect can cause image distortion, disrupt signal transmission, and compromise the accuracy of the analytical measurements made within these devices.

Bubbles formed during the PDMS mixing process are typically removed through a process known as degassing.^7,8^ The mixture is placed in a vacuum chamber, creating a negative gauge pressure environment. Upon reduction of pressure, entrapped air bubbles within the PDMS mixture undergo volumetric expansion and migrate to the surface.^9^ Concurrently, the diminished pressure facilitates the escape of air bubbles from the PDMS mixture. The vacuum is sustained for a specific time, typically ranging from several minutes to a few hours, contingent on the quantity and consistency of the PDMS mixture. Centrifugation is also employed for the removal of bubbles from PDMS mixtures, offering an alternative approach to degassing.^10,11^ Due to the differences in density between the PDMS matrix and the air bubbles, the bubbles experience a buoyant force pushing them toward the rotational axis. As the centrifuge continues to spin, the bubbles gradually migrate toward the free surface interface, forming a distinct layer of accumulated bubbles.

Apart from centrifugation, a churning motion can also be implemented to remove bubbles from the PDMS mixture. The churning motion is used in various separation processes in different industries. One of the notable areas where it is used is dairy processing.^12^ Cream separators utilize a churning motion to separate the lighter cream from the denser milk through centrifugal force generated by high-speed rotation. The churning action causes the denser milk to move toward the outer edges of the separator, while the lighter cream collects in the center. Churning motion is also employed in separating emulsions and suspensions, such as in the pharmaceutical and chemical industries, where mixing and agitation are crucial for promoting phase separation. Even though the efficacy of the churning process is known, it is not yet used for bubble removal in PDMS mixtures.

In this work, we explore the possibilities of utilizing the churning motion for bubble removal from PDMS mixtures. A simple nonelectric and low-cost churning device of do-it-yourself (DIY) type is developed for this purpose. The hydrodynamics of bubble removal is studied while applying churning motion. Here, the PDMS mixture is systematically subjected to an alternating clockwise and anticlockwise rotational motion. The efficacy of bubble removal during the churning process is studied by using images obtained from the front and top views. Quantification of bubble removal is made from image analysis. A growing body of literature suggest that do-it-yourself approaches can be leveraged for thread & paper-based microfluidic applications.

Two PDMS mixtures, prepared with base polymer to curing agent ratios of 10:1 and 10:2, respectively, are considered for the study. These two ratios are commonly used in the preparation of microfluidic channels or devices in soft lithography. The 10:1 mixture results in a softer polymer upon curing, while the 10:2 mixture yields a harder one. The PDMS mixtures are subjected to fast-, medium-, and slow-paced churning motion, and the percentage of bubbles removed for varying churning duration is studied. The efficacy of bubble removal by churning is later compared to that of the traditional vacuum degassing technique.

Details of the experimental setup used for the visualization study and the methodology used to quantify the percentage of bubbles removed due to churning motion are explained in the next sections.

## 2 Experimental Setup

**Figure 1:**
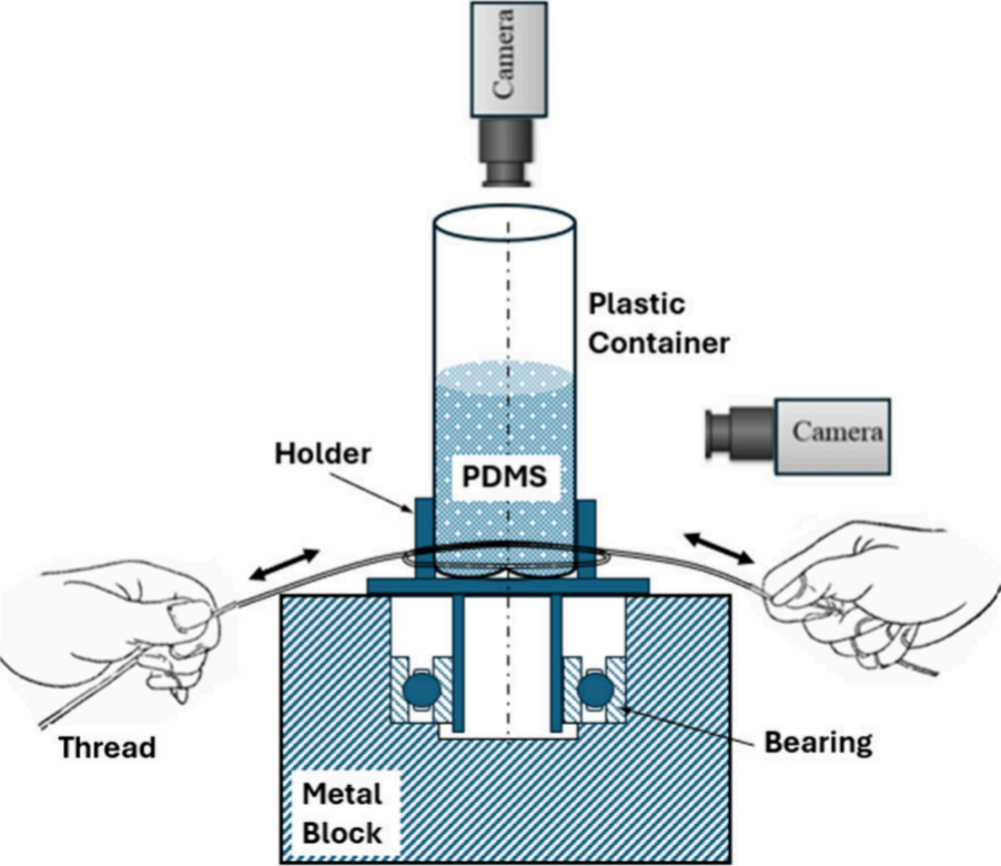
Schematic sectional view of the experimental setup.

The schematic sectional view of the low-cost DIY churning device used in this study is shown in Figure 1. The device is designed to be operated manually without relying on electrical power sources. It consists of a metal block, which forms the base and supports the device during the churning process. A hole is drilled in the metal block to fix the outer ring of the ball bearing with a tight fit. A holder for the plastic container fabricated from poly(vinyl chloride) material is fixed tightly to the inner ring of the bearing, as shown in Figure 1. The transparent plastic container containing PDMS mixture is then rotated using a thread wound around the holder. The to-and-fro motion of hands churns the mixture due to alternating clockwise and anticlockwise rotation of the container.

Three different churning speeds are used in the study. The churning speeds are estimated by recording the number of to-and-fro motions per unit time. The to-and-fro cycles per unit time determine the churning frequency (*f*). Based on the pull-stroke length (*L* = 700 mm) and the outer diameter of the holder (*D* = 48 mm), the angular velocity (ω) is estimated using the following equation.
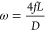
1The established churning speeds are listed in Table 1. The indicated speed ranges can be achieved manually and consistently.

**Table 1:** List of Different Churning Speeds Used in the Study

Sl. No.	Churning speed	Approximate angular velocity, ω (rad/s)
1	Slow	75 ± 5
2	Medium	115 ± 5
3	Fast	135 ± 5

Two PDMS mixtures (Base polymer + Curing agent), 10:1 and 10:2, are used to investigate the efficacy of bubble removal by the churning process. For the formulation of the PDMS mixture, a base polymer of silicone is utilized in conjunction with a curing agent, specifically a silicone resin solution containing methylvinylcyclosiloxane (SYLGARD 182 Silicone Elastomer, supplied by Dow Europe GMBH and Dow Silicones Deutschland GMBH, Germany). Approximately 50 mL of the mixture is prepared in a plastic container and placed in the holder. It is ensured that the mixture has a sufficiently large number of bubbles before the start of the experiment. The mixture is then churned at different (slow, medium, and high) speeds by pulling the thread wound around the holder. The front (side) and top view images are captured using two digital microscope cameras (Dino-Lite Digital Microscope, Ammo Electronics Corporation, Taiwan). The maximum duration of churning time applied is 420 s for slow-speed churning.

As the study aims to examine the effect of the PDMS viscosity on the bubble removal rate, two PDMS mixtures are considered. The dynamic viscosity value of the 10:1 PDMS mixture is 3000 cP, which is higher than the 2100 cP viscosity value for the 10:2 PDMS mixture.^13^ Three distinct churning speeds (Table 1) are applied to each of these PDMS mixtures, and the images are captured during the churning process. From the images obtained during experimentation, the percentage reduction in bubbles is estimated. The methodology used for this analysis is explained next.

## 3 Methodology

To quantify bubble removal, images captured during the churning process are analyzed. Since our primary goal is to investigate the effectiveness of churning motion in facilitating bubble removal, an approximate quantification approach is adopted in the study. Emphasis is placed on the demonstration of churning motion’s capability to remove bubbles from PDMS samples using a DIY device. To ensure the reliability of the results and to minimize systematic errors caused by initial bubble distribution in the samples, two specific approaches are implemented: First, bubbles are introduced into the PDMS mixture through a mechanical agitation process. This process is standardized with a fixed number of agitation strokes applied for 20 s across all experimental trials to achieve a similar bubble density. Second, multiple trials are conducted to estimate experimental deviations accurately, and it is observed that despite minor variations in initial bubble distribution within the samples, the results are consistently similar. The initial bubble distribution thus created serves as the baseline condition for all experiments.

According to the methods described in the Experimental Setup section, images of the front and top views are taken at 30 s intervals during the churning. The mixture, kept in a container, is subjected to churning at slow, medium, and fast speeds. These images, captured throughout the churning process, are postprocessed and analyzed to quantify the percentage of bubble reduction.

In the captured images, areas occupied by bubbles appear white, while regions filled with clear PDMS are shown as black. This contrast allows for precisely quantifying gas bubbles within the mixture by analyzing the area covered by white pixels. Initially, RGB-colored images are converted into binary images during postprocessing. In these binary images, a pixel value of one indicates a white region containing bubbles, whereas a value of zero indicates a black region devoid of bubbles. The percentage reduction of bubbles caused by churning is estimated for each image by using the following equations.
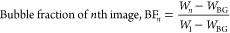
2
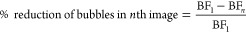
3Here, *W* represents the number of pixels in the image with a value of one. The subscripts, *n*, 1, and BG, represent the *n*th, first, and background image, respectively.

**Figure 2:**
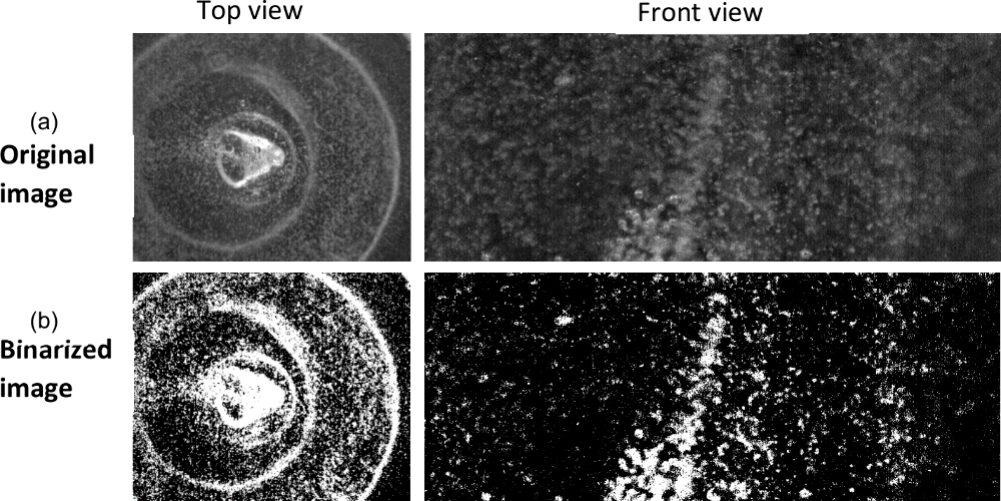
Image postprocessing for evaluating the percentage of bubbles from top and side view images: (a) original image and (b) binarized image.

As shown in the above equation, the number of white pixels resulting from the background was considered when estimating the bubble fraction in an image. The number of white pixels formed by the background was initially estimated from the image, with a clear PDMS mixture having no bubbles inside. During imaging, black paper was placed behind the sample to get a black background. This helped to reduce the background effects; however, in the top view image, the reduction of background effects using black paper was marginal. This is evident from the sample image shown in Figure 2. where, the original and the postprocessed images obtained during the churning process are depicted. The front view image appears clear of any background effects when binarized, with only minor effects of light reflection caused by the curved shape of the sample container. However, the top view presented a challenge due to the conically inward-protruding base of the plastic container, resulting in a background with more white pixels. Thus, a correction for background effects in quantifying the bubbles, as given in eq 1, is required, especially for top-view images. It is worth noting that top-view images may not quantify all bubbles accurately as some may be concealed within the background. Nevertheless, the background is subtracted in all cases, which means that the quantity of bubbles concealed by the background can be viewed as a systematic bias that will not impact comparative observations.

## 4 Results and Discussion

After the background effects in the captured images were corrected, the percentage reduction in bubbles caused by churning is evaluated. Observations for various cases conducted in the study are provided in this section. Observations for 10:1 PDMS mixtures are discussed first, followed by their comparison with those of the 10:2 PDMS mixture. The effect of churning speed and time are analyzed. To ensure the accuracy of the results, three repetitions of the experiments are conducted. Based on these repetitions, experimental uncertainties are estimated.

**Figure 3:**
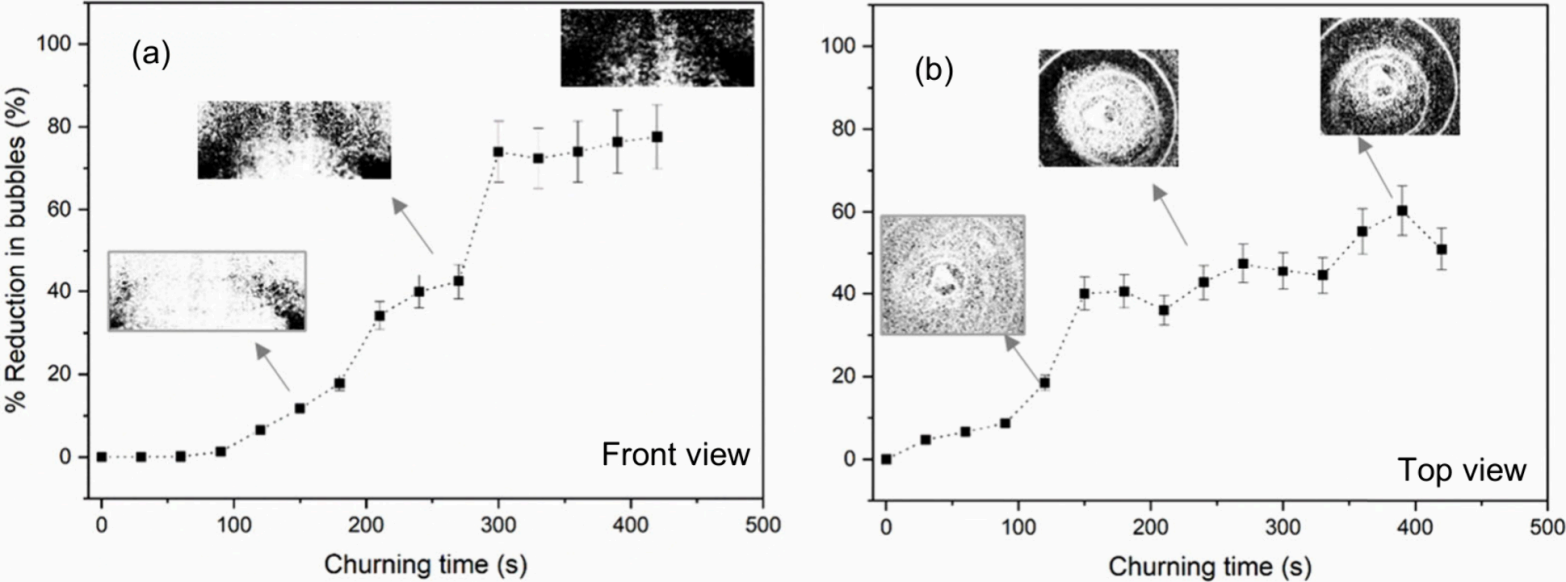
Percentage bubble reduction observed while applying slow-speed churning for the 10:1 PDMS mixture. Estimation from (a) front view and (b) top view.

In Figure 3, the graph plots the percentage reduction in bubbles against churning time for a 10:1 PDMS mixture while applying churning at a slow-speed (75 ± 5 rad/s). Images are captured every 30 s of churning time. Equation 2 is used to quantify the percentage reduction in bubbles observed from the images. Estimations obtained from the front and top views are shown in Figure 3(a) and Figure 3(b), respectively. Sample images obtained at different churning times are listed in the inset. The percentage reduction of bubbles from the front and top views shows a similar trend. Slow-speed churning for at least 100 s is required to obtain a noticeable reduction in bubbles. Moreover, it is observed that a 10:1 PDMS mixture demands more than 300 s of churning to achieve a minimum of 50% reduction in bubbles. A 100% bubble removal is not achieved with a slow churning speed, even after a significant amount of time.

**Figure 4:**
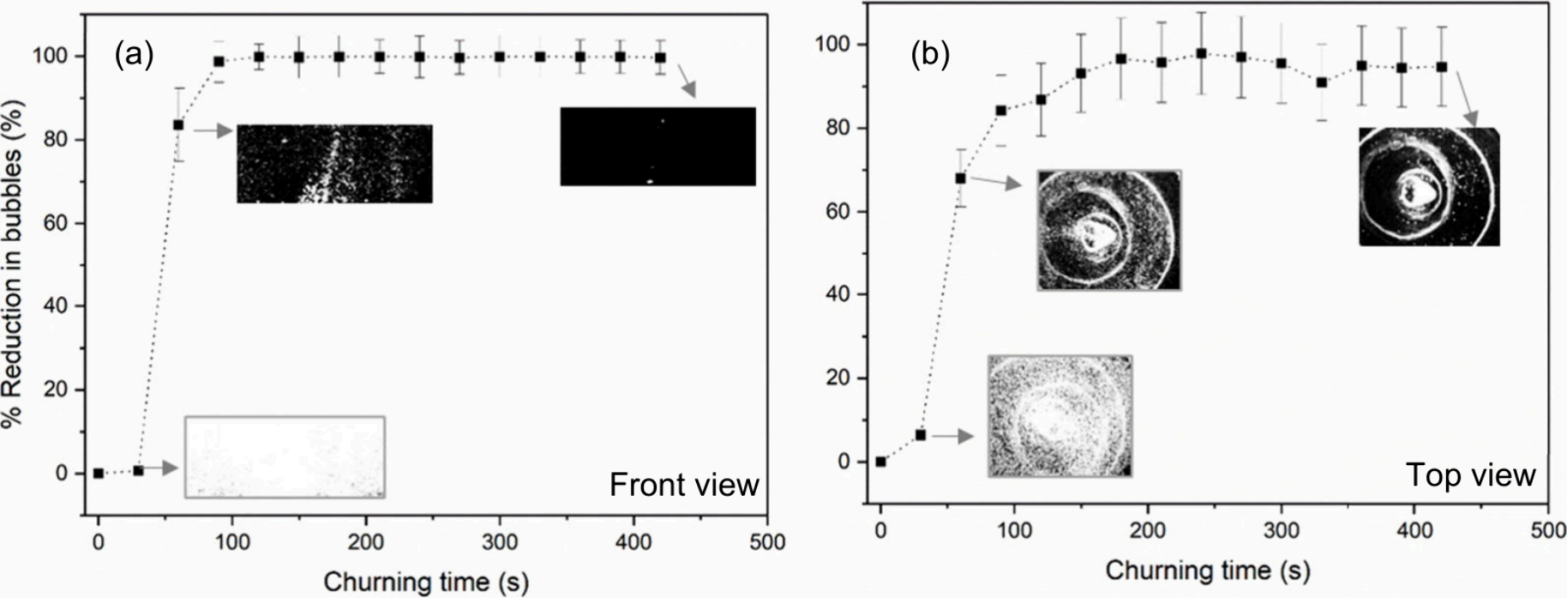
Percentage bubble reduction observed while applying medium-speed churning for the 10:1 PDMS mixture. Estimation from (a) front view and (b) top view.

Figures 4(a) and (b) illustrate the reduction in bubbles from the front view and top view for the 10:1 PDMS mixture as the churning speed is increased to medium (115 ± 5 rad/s). Unlike the case with slow-speed churning, with medium-speed churning, a rapid reduction in bubbles is observed. More than 50% reduction in bubbles is observed within a churning time of 100 s with medium-speed. Incorporating medium-speed churning into the process resulted in the removal of nearly 100% of the bubbles.

**Figure 5:**
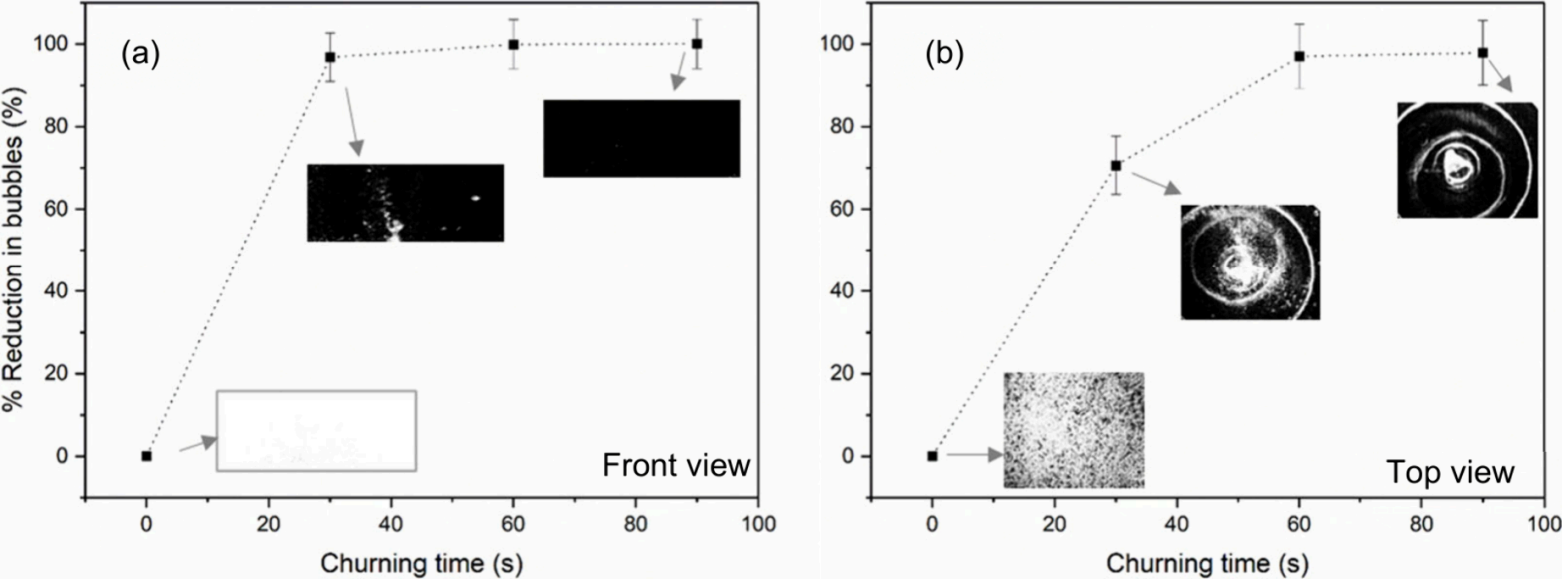
Percentage bubble reduction observed while applying fast-speed churning for the 10:1 PDMS mixture. Estimation from (a) front view and (b) top view.

Figures 5(a) and (b) display the reduction of bubbles while fast-speed churning (135 ± 5 rad/s) is applied for the 10:1 PDMS mixture. The front and top views are estimated and shown, respectively. Implementing a fast-speed churning process is noted to provide a more expedient removal of the bubbles. Specifically, within a brief period of 30 s, 50% of the bubbles are effectively removed through this technique. Additionally, it is observed that nearly all bubbles are eliminated within the initial 60 s of using the fast churning.

**Figure 6:**
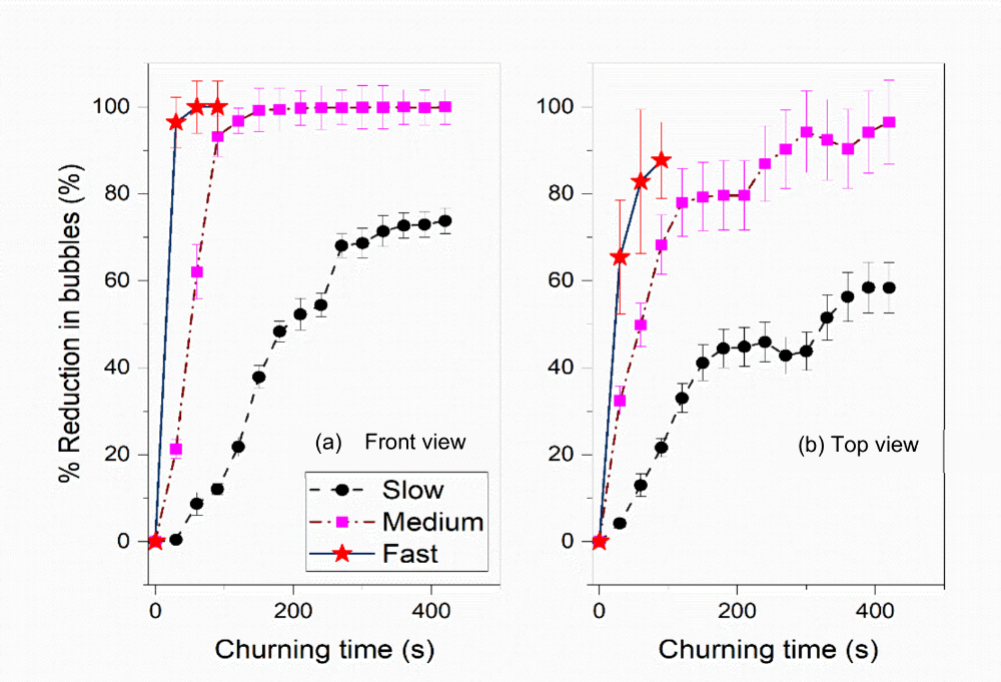
Percentage bubble reduction observed while applying slow-, medium-, and fast-speed churning for the 10:2 PDMS mixture derived from (a) the front view and (b) the top view.

After conducting experiments with a 10:1 PDMS mixture, the percentage reduction in bubbles is quantified for 10:2 PDMS mixtures. Figures 6(a) and (b) depict the percentage reduction in bubbles with time for 10:2 PDMS mixtures at three different churning speeds, as observed from the front and top perspectives, respectively. The results showed that fast churning was significantly more effective in removing bubbles for the 10:2 PDMS mixture than medium and slow churning. On average, a 50% reduction in bubbles was observed at approximately 200, 100, and 20 s for slow, medium, and fast churning speeds, respectively. When Figures 6(a) and (b) are compared, bubble removal is more pronounced in the front view than in the top view. This is because the centrifugal force from the PDMS liquid during the churning process moves the bubbles toward the center of the container and to the top free surface. As the mixture is churned, the denser liquid (PDMS) is pushed toward the outer peripheral surface of the container while the less dense air bubbles move toward the center. Over time, the bubbles begin to merge and rise to the top due to their buoyancy.

**Figure 7:**
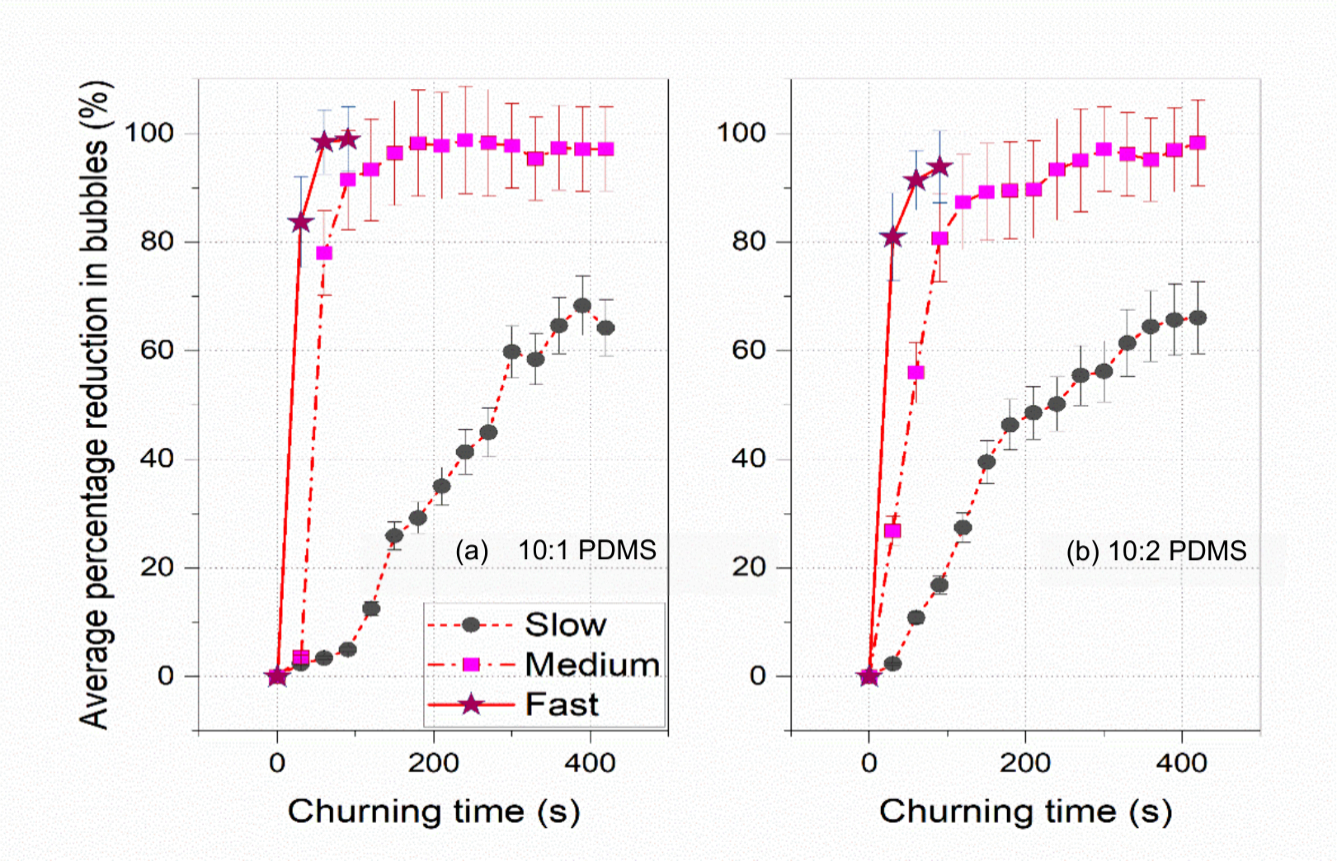
Averaged percentage bubble reduction observed while applying slow-, medium-, and fast-speed churning for (a) a 10:1 PDMS mixture and (b) a 10:2 PDMS mixture.

Figure 7 plots the average value of the percentage reduction obtained from the front view and top view observations for varying churning times. Figure 7(a) shows the averaged percentage reduction values for the 10:1 PDMS mixture, whereas (b) shows the same for the 10:2 PDMS mixture. Based on the averaged values (based on averaged values of front and top views), it has been observed that fast churning outperforms medium and slow churning in removing bubbles from PDMS mixtures. Although there is a similar trend in bubble removal for 10:1 and 10:2 PDMS mixtures, it is more efficient for 10:1 PDMS, particularly for fast and medium-paced churning. This is especially evident from the medium churning curve, where the 10:1 PDMS mixture achieves almost 100% bubble removal faster than the 10:2 PDMS mixture. This observation may be attributed to the higher viscosity value of the 10:1 PDMS mixture compared to the 10:2 PDMS mixture.^13^

**Figure 8:**
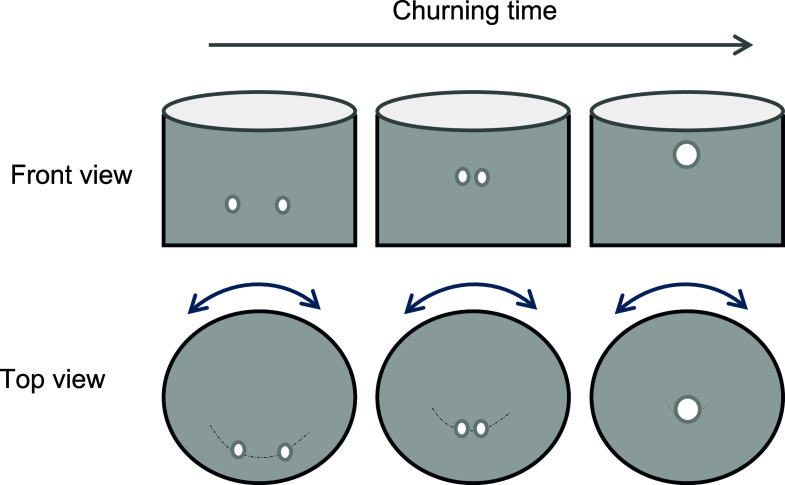
Bubble dynamics while applying churning.

Upon comparison of the curves depicted in Figures 6(a) and (b), it can be observed that the bubbles are disappearing more prominently from the front view, as opposed to the top view. This is because bubbles are pushed toward the center and top free surface. A plausible mechanism of the bubble removal process during a churning motion is depicted in Figure 8. For simplicity of explanation, the motion of two bubbles close to each other is only considered. The illustrations show the different locations of the bubbles as they move within the container at various intervals of churning. During the churning motion, the container holding the sample is rotated clockwise and anticlockwise such that applied angular velocity values undergo a sinusoidal pattern, reaching a peak velocity at which the direction of rotation is changed. As shown in the front view, the bubbles are pushed toward each other during the alternating clockwise and anticlockwise rotation. In addition, due to the vortex created by rotation, the bubbles tend to move inward and upward. As the bubbles move close to each other, they can coalesce or bounce back, depending on the approach velocity. An earlier study demonstrated that the likelihood of bubble coalescence increases with decreasing approach velocities.^14^ Specifically, bubbles approaching each other at lower speeds are inclined to merge, whereas those approaching at higher speeds tend to collide and deflect.^15^ Following coalescence, the removal of bubbles during the churning process is expedited, attributed to the enhanced upward buoyant force acting upon a larger bubble compared with a smaller one. Concurrently, bubbles are propelled toward the container’s center due to the centrifugal force generated as the liquid moves outward, thereby inducing an inward movement of the bubbles. The overall bubble movement involves the interplay between approach velocity, coalescence, and the resultant dynamics within a churning liquid medium.

**Figure 9:**
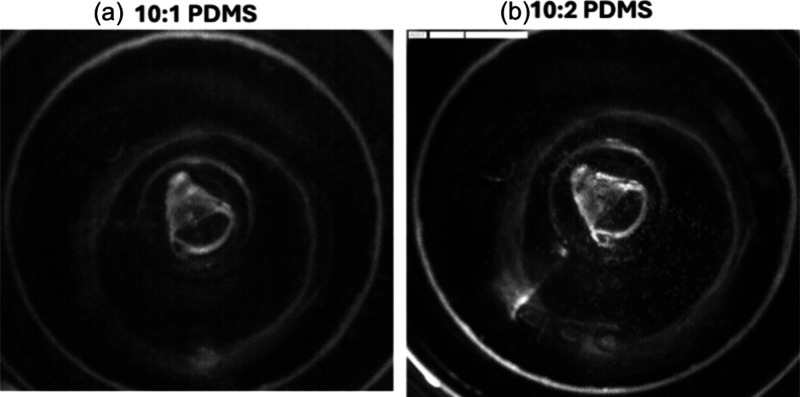
Top view observation after 90 s of fast churning for (a) a 10:1 PDMS mixture and (b) a 10:2 PDMS mixture.

Observations from Figure 7 indicate that the efficiency of bubble removal is higher for the 10:1 PDMS mixture than for the 10:2 PDMS mixture. This can be attributed to the higher viscosity value of the former mixture. Higher viscosity would impart resistance to the bubble motion, reducing their approach velocities and facilitating coalescence. Since bubbles are eventually released from the top surface, comparing the top view for the two mixtures would be ideal. The top view of the container after churning at high speed for 90 s for 10:1 and 10:2 PDMS mixtures are depicted in Figures 9(a) and (b), respectively. Even though bubbles are negligible in the front view, more bubbles are visible in the top view for the case with 10:2 PDMS mixtures. This may be because 10:2 PDMS mixtures have lower viscosities, which would result in lesser coalescence when compared to 10:1 PDMS mixtures and their subsequent removal from the top free surface of the container.

**Figure 10:**
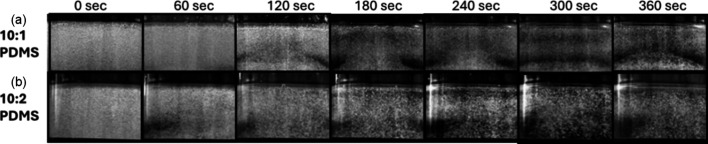
Time variant front view observation with slow-speed churning for (a) a 10:1 PDMS mixture and (b) a 10:2 PDMS mixture.

**Figure 11:**
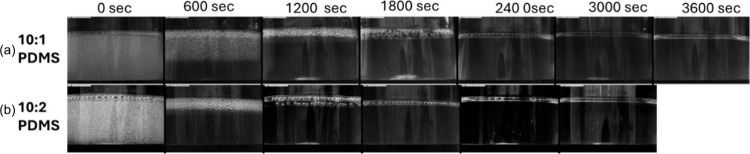
Time variant front view observation with vacuum degassing for (a) a 10:1 PDMS mixture and (b) a 10:2 PDMS mixture.

Another effect caused by the viscosity of the PDMS mixture is displayed in Figure 10. Here, observations from churning at a slow-speed are compared. The images display the front views of 10:1 and 10:2 PDMS mixtures at different churning intervals. Initially, both mixtures had equal amounts of bubbles dispersed in them. As churning progresses (at *t* = 360 s), a clear separation shear layer is visible for the 10:1 PDMS mixture. However, such a separation zone is not distinctly visible for the 10:2 mixture. This is probably created because of the more viscous nature of the 10:1 PDMS mixture, making it move together with the wall (no-slip motion). The separation layer is produced by the combined effect of shear and the shape of the container (inward protruding base). For the 10:2 PDMS mixture, the effect of moving walls and the vortex shape created inside the container due to churning is not as visible as for the 10:1 PDMS. The figure also shows that a slow churning process was not efficient in removing the bubbles from either of the mixtures,

Finally, the efficacy of the churning mechanism is compared to the existing vacuum degassing technique used for removing bubbles from PDMS mixtures. The current technique to remove bubbles from PDMS involves keeping the mixture inside a vacuum desiccator chamber until all of the bubbles are cleared. The negative gauge pressure is applied on the mixture’s free surface to help remove the bubbles. For this study, 10:1 and 10:2 PDMS mixtures are initially agitated to saturate them with bubbles and subjected to a vacuum pressure of 100 kPa. The front view of the mixture taken at intervals of 10 min while applying the vacuum is presented in Figure 11. The mixture is removed from the vacuum chamber and photographed to assess the impact of the degassing process on bubble elimination. It is observed that degassing for one h completely removes bubbles in the 10:1 PDMS mixture, whereas in the 10:2 mixture, only 50 min of degassing is needed. Vacuum degassing is observed to be more effective in removing bubbles in a 10:2 PDMS mixture than in a 10:1 PDMS mixture. This efficiency is attributed to the mechanism of vacuum degassing, which extracts bubbles through a pressure difference, with less viscous fluids presenting less resistance to the applied negative pressure.

In comparison to vacuum degassing, the churning process is a faster method to remove bubbles from PDMS mixtures. The results of the study showed that the complete removal of bubbles took less than 100 s with fast churning, while it took over 3000 s with vacuum degassing. This indicates that the churning motion offers a simple, efficient, and cost-effective alternative to the existing vacuum degassing technique.

Churning offers several advantages over rotation methods, such as centrifugation for bubble removal in PDMS mixtures. It is a simple method that can be used by laboratories with limited resources. It is also compatible with larger volumes, making it versatile in handling diverse sample sizes. Moreover, churning enables researchers to control the mixing parameters precisely, tailoring the agitation intensity and duration to suit specific PDMS formulations.

## 5 Conclusions

In this study, a low-cost, hand-operated, DIY churning device is developed to degas PDMS mixtures. The effectiveness of bubble removal from PDMS mixtures through churning is assessed using an approximate quantification approach. Analysis of the images captured during the churning process is used for that purpose. The hydrodynamics involved in the removal of entrapped bubbles from PDMS mixtures through the application of periodic churning motion involving both clockwise and anticlockwise rotations is also explored. Two commonly used PDMS mixtures with base polymer to curing agent ratios of 10:1 and 10:2 are investigated, noting that the mixture with the 10:1 ratio exhibits higher viscosity than its 10:2 counterpart. These mixtures are subjected to varying speeds of churning motion—fast, medium, and slow—and the impact of these varying speeds on the percentage of bubbles removed over different churning durations is studied.

The following are the salient observations from the study.

- During the churning process, the percentage of bubbles removed is more noticeable from the front than the top view. This is because the centrifugal force pushes the bubbles toward the center of the container, eventually causing them to escape to the top free surface due to buoyancy.
- Slow-speed churning of the 10:1 PDMS mixture requires over 300 s to achieve a 50% bubble reduction, while medium-speed churning accomplishes the same within a 100-s time frame. Fast-speed churning proves to be the most effective, achieving more than a 50% reduction in just 30 s and nearly complete elimination within 60 s.
- A fast-speed churning is observed to be highly effective in degassing PDMS mixtures. Regardless of viscosity, fast-speed churning outperforms medium and slow churning for both the PDMS mixtures.
- Although the percentage of bubble removal trend is similar for the 10:1 and 10:2 PDMS mixtures, the former proves more efficient due to its higher viscosity. The increased viscosity lowers the approach velocity of the bubbles and facilitates coalescence, making the process of removing bubbles more effective.
- The churning process removes bubbles from PDMS mixtures more quickly than the traditionally used vacuum degassing technique. For the 10:1 PDMS mixture, complete bubble removal takes less than 100 s with fast-speed churning, compared to over 3000 s with vacuum degassing.

Overall, the churning process appears to be a viable alternative to the vacuum degassing technique currently employed in laboratories. It is a simple and low-cost method that does not require any specialized equipment or expertise, making it an attractive option for researchers and professionals in the field.
